# Which Journal?

**Published:** 1991-09

**Authors:** Mike Read


### WHICH JOURNAL?

It is the time of year when Medical Librarians issue the annual plea for a review of the journals taken by the library. With the ever present pressures of a budget and the ever increasing cost of subscriptions it is understandably important that only essential journals are taken. Indeed the problem is compounded by the explosion in the number of new titles all claiming to fill an important gap in the present spectrum. From the Publisher's point of view this can be very lucrative and there is no shortage of people willing to submit articles, as long as the pressures for Registrars and Senior Registrars to have papers published to pad out their CV continues. How then can we decide which journals are required? In order to stretch the library budget as far as possible it is common practice to ask individuals who subscribe to a journal to pass the journals on to the library but this by no means solves the problem.

A number of pharmaceutical companies now offer regular digests for different specialities or topics where all the journals are scanned and abstracts of relevant articles are printed. This is a very convenient way of keeping up to date with what is happening and the companies are usually more than happy to assist with the provision of reprints, particularly if it is an area which could increase their sales.

The interest of consumer groups has done a lot to simplify our choice of which journals to take and it is now very simple to select journals which keep on up to date, keeping abreast of new developments and at the same time telling us what the public are hearing about and journals which one can guarantee will be read thoroughly from cover to cover, ensuring maximum value for money. It is a common sight now to see patients coming into the Out patients Clinic holding a photocopy of an article they have seen in one of these journals.

All, therefore, that is necessary for the medical librarian to do to provide us with this quality service and at the same time keeping within the budget is:—

a) Encourage people to pass on their own journals.
b) Encourage the wider use of pharmaceutical industry digests.
c) Subscribe to the 3 journals.

What are these journals? Bella, Best and Women's World.

Mike Read

